# Industry perspective: the gentle bear claw at work

**DOI:** 10.1016/j.igie.2023.10.009

**Published:** 2023-10-23

**Authors:** Marc Schurr, Geoffrey Yates, Linda S. Lee

**Affiliations:** 1 Ovesco Endoscopy AG, Tübingen, Germany; 2 Geoffrey Yates, Ovesco Endoscopy USA, Inc, Cary, North Carolina, USA; 3 Brigham and Women's Hospital, Boston, Massachusetts, USA

## Editor’s Introduction

**Figure 1:**
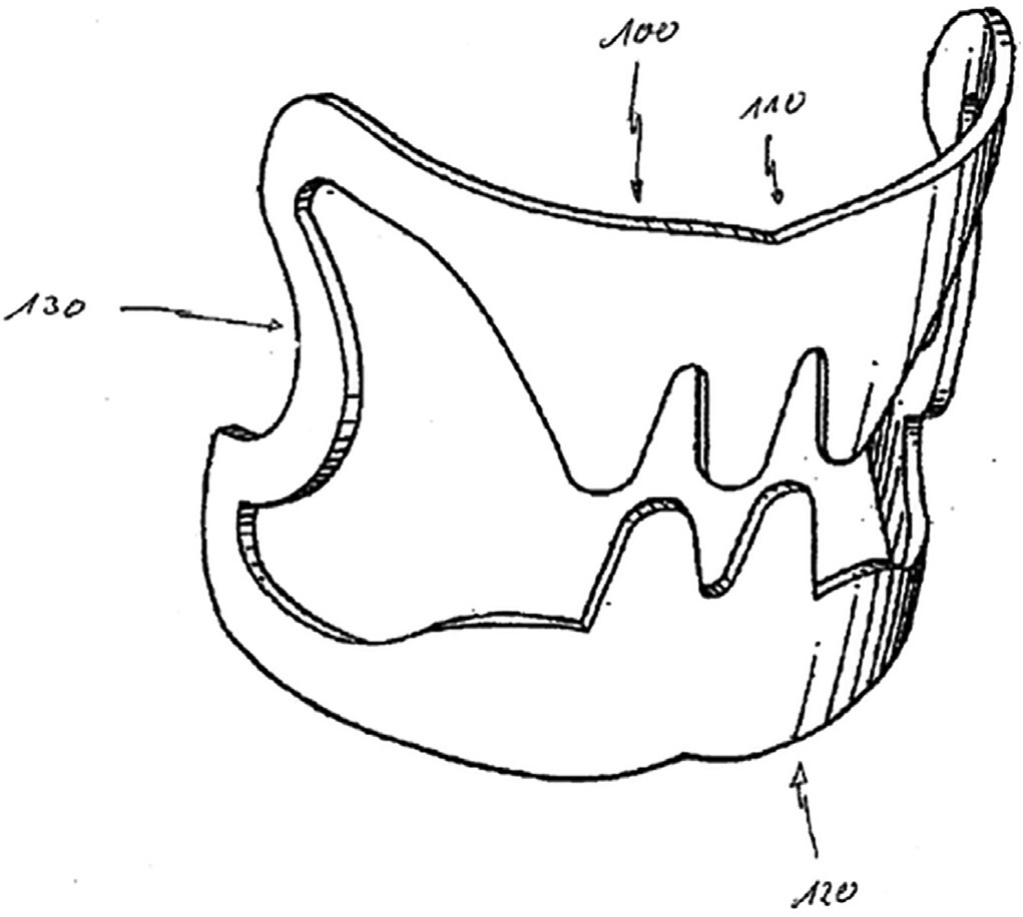
Drawing of over-the-scope clip for U.S. patent application.

Through-the-scope metal clips for endoscopic hemostasis were introduced in 1975 by Hayashi et al^1^ in Japan. However, issues with the complicated technique, suboptimal retention, and potential for tissue injury prevented widespread dissemination of this procedure until further improvements were made in the late 1980s by Hachisu and his team working with Olympus Optical Co.^2^ His study in 51 patients noted 84.3% with “permanent hemostasis” after 17.4% of patients required a second procedure with repeat clipping and that the “clamping characteristics were similar to hemostasis by surgical ligation.” This foreshadows the development of the OTSC Clip by Ovesco Endoscopy (Cary, NC, USA), which was designed to mimic surgical suturing and received U.S. Food and Drug Administration (FDA) 510(k) clearance in December 2010 (Fig. 1).^3^ The Padlock clip by Steris Medical (Mentor, Ohio, USA) is the other over-the-scope device on the market, which received FDA 510(k) clearance in April 2018. Interestingly, despite further advances in the through-the-scope clip technology with rotation and reopening capabilities, studies have not confirmed the superiority of these clips compared with thermal therapy for treating nonvariceal upper GI bleeding. The Ovesco OTSCs hold promise for treating recurrent bleeding in peptic ulcer disease and may lead to decreased recurrent bleeding when used as primary treatment, with several randomized controlled trials reporting this outcome in patients at high risk for recurrent bleeding.^4^, ^5^, ^6^, ^7^, ^8^

It is my pleasure to discuss the journey of OTSC and Ovesco with Dr Marc Schurr and Geoffrey Yates. Marc founded Ovesco in 2008 and has been Chief Executive Officer since 2021. Marc successfully led Ovesco’s technology and product development, clinical science, and international market launch. Before starting Ovesco, Marc held various positions in academia, contract research and consulting, and became a serial entrepreneur, creating an incubator for medtech products and services in Tübingen, Germany. He joined Steinbeis University Berlin in 2005, where he had multiple teaching and scientific responsibilities as director of the IHCI Institute until 2021. He serves on the board of different institutions and committees in industry, medicine, and the investment community as well as for charitable societies. Marc graduated as a medical doctor from Eberhard Karls University School of Medicine in Tübingen and has a medical science degree from the same institution. He also holds an executive Master of Business Administration from École Supérieure de Commerce de Paris.

Geoffrey Yates has served as President of Ovesco Endoscopy USA since its founding in 2010. He has extensive experience in sales of innovative medical devices, building international distribution channels, marketing, and product development. Before joining Ovesco, he worked at several companies, including Boston Scientific, Smith and Nephew Endoscopy, Abbott Vascular, Bard Electrophysiology, and a number of startup companies. Geoffrey is a member of Ovesco's Executive Board and is also responsible for Ovesco Endoscopy’s U.S. business. He is a graduate of the University of Cincinnati and has a Bachelor of Science degree in Business Administration.

Section Editor: Linda S. Lee, MD

**Figure 2:**
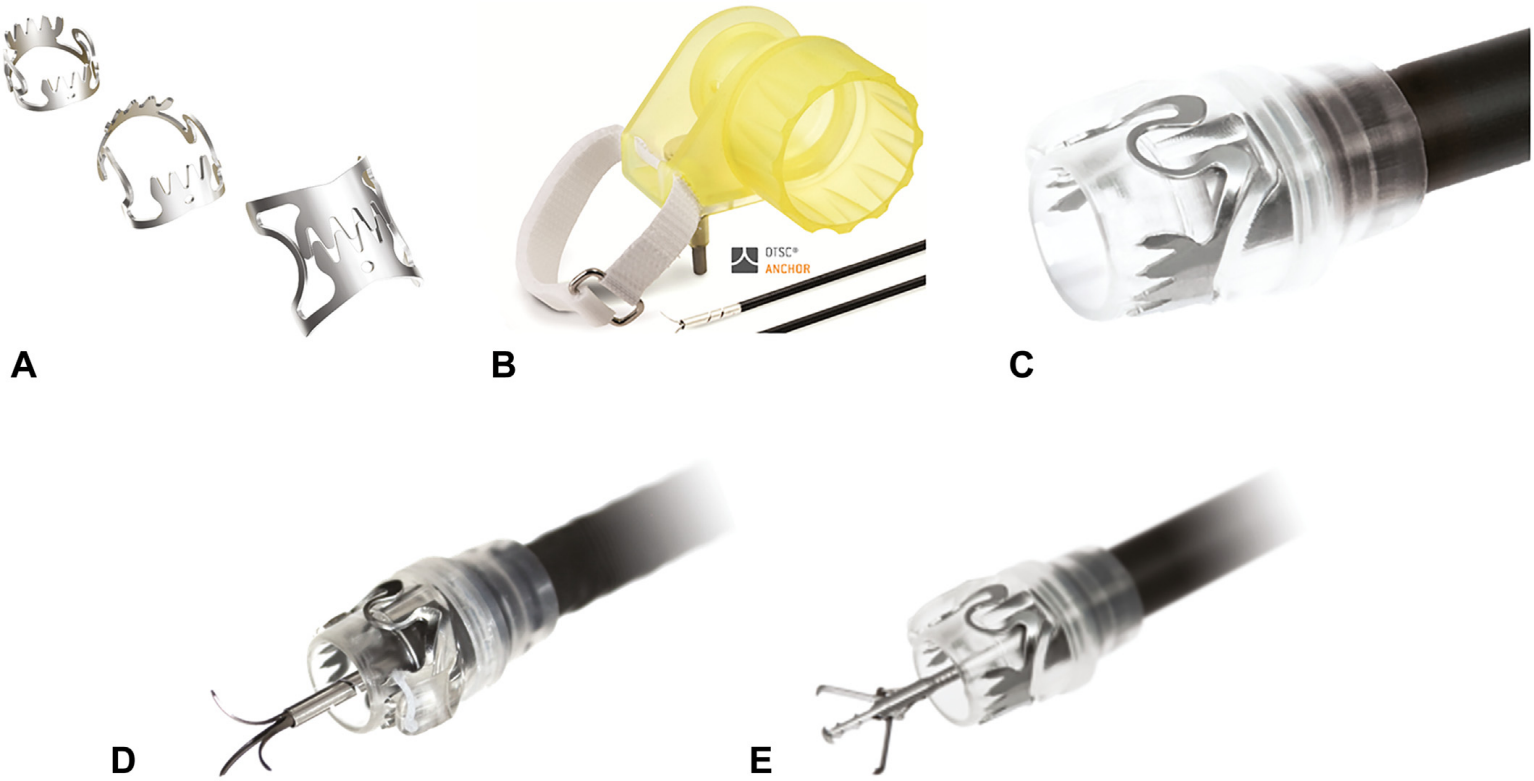
**A,** Over-the-scope clip (OTSC) with rounded teeth (type a). **B,** Handwheel. **C,** OTSC on cap. **D,** OTSC anchor. **E,** OTSC twin grasper.

**Linda Lee (LL): Thank you very much for making time with your incredibly busy schedules to discuss the evolution of the OTSC in GI. Would you discuss how this novel idea of an OTSC came about and became the focus for your company?** (Fig. 2)

**Marc Schurr (MS):** It has been an amazing journey. The fundamental idea was to mimic surgical suturing endoscopically, doing a U-stitch suture or partial stapling by means of a flexible endoscope. The original idea was that OTSC would be a hemostatic device which compresses an ulcer similar to how a suture would treat it. Because suturing is difficult with a flexible endoscope, we sought to do something that emulates the compression done by suturing but by using a clip. The clip cannot run through the working channel because it would be too small. So, the way it would be installed would be like band ligators for variceal bleeding. If you look at the way an OTSC compresses the tissue, it is like a suture line because of the zigzag shape that the teeth produce in the tissue. Although nitinol is pretty standard in endoscopic devices now, it was a novel material at that time, which opened enormous new opportunities in medical devices. Bending a steel clip would be difficult by means of forced deliveries along the shaft of a flexible endoscope so you would need to store the energy that needed to be deployed into the tissue to compress the tissue in the material itself. That is only possible with nitinol because only nitinol can be deformed so much and go back to its original shape using the shape memory effect. In short, the idea was to have something as effective as a U-stitch through an ulcer and at the same time leverage the potential of the superelastic alloy to produce the compression force that would be needed to do so. This is a bit of a technical answer, but the original idea was a research project, a technical exercise.

**Geoffrey Yates (GY):** My original background was at Ethicon, Johnson & Johnson, and I was involved in the stapling devices back then. I use this analogy with OTSC. We use the porcine stomach a lot for training on OTSC. When you deploy the clip, you see the ball of tissue on top of the clip, but in the animal lab, I like to invert the stomach and show the other side. You will see the clip on the other side, and it looks more like a surgical staple line. I would tell the gastroenterologist that this is what the surgeon would see on this side of the clip because you will see the compression force. I wish we could have called it a clamp rather than a clip but that was an FDA issue comparing it more to traditional clips, although it is more like a surgical clamp.

**Figure 3:**
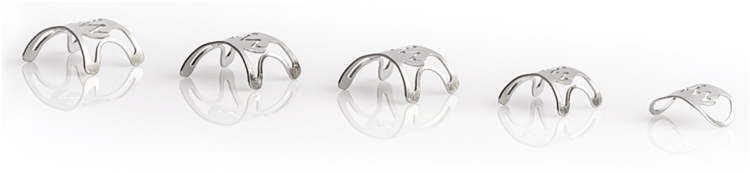
Over-the-scope clip type 12 gc, t, a, mini, and stentfix (left to right).

**LL: There are so many different shapes and types of teeth. Would you talk a bit about how all these different things came to being? (** Fig. 3 **)**

**MS:** Another aspect of the clip is that the shape of the teeth coming together keeps space between the teeth free, which ensures that the microperfusion of blood to the tissue remains intact. Otherwise, there would be necrosis of the tissue. It is similar to a staple line, which also does not lock down completely but leaves space between the staples. The same applies here, and this ensures that the tissue remains vital and heals. Earlier on, the original clip had blunt, round teeth. Then, because everyone was interested in closure for transluminal access to the abdominal cavity, we modified the clip with little spikes to hold the wall even more firmly, which we call “t” clips. This is why we have “a” and “t” clips: a for compression only and t for closure. That became a second variation. From there, we worked our way to more applications such as stent fixation clip and full-thickness resection. It is a platform technology that we modified into further derivatives of the original technology to meet the demands for new applications.

**GY:** Marc and I talked about different versions of the clip. We also have the mini now and “gc” for gastric closures specifically. We then evolved into the full-thickness resection devices that Marc mentioned.

**Figure 4:**
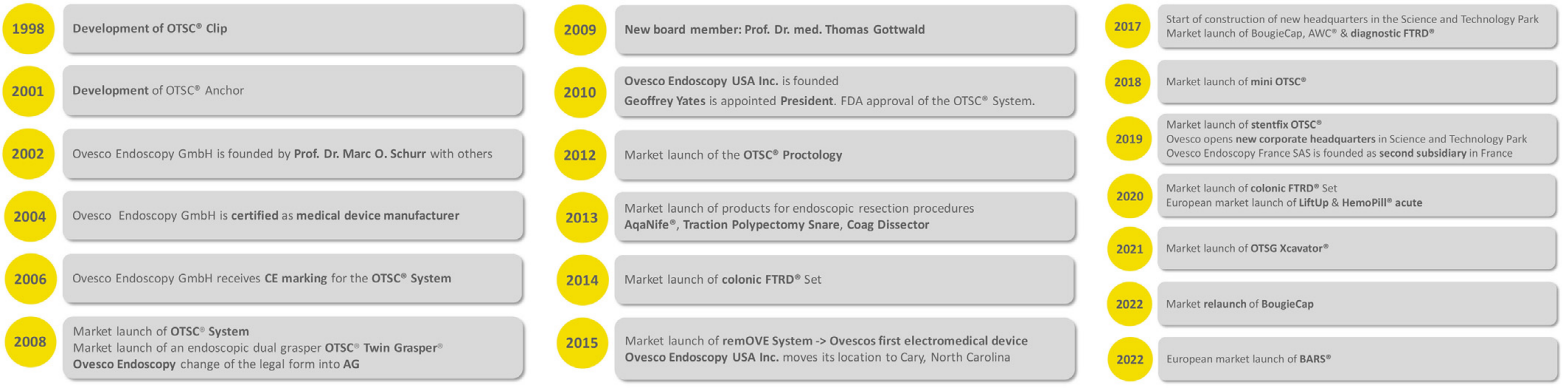
History of Ovesco Endoscopy. *FDA*, U.S. Food and Drug Administration.

**LL: Would you discuss the key steps in taking the wonderful idea of OTSC to something we can actually use in people? (** Fig. 4 **)**

**GY:** The timeline started with the CE mark in April 2009. We received 510(k) clearance from the FDA in December 2010 and sold our first OTSC in the United States in January 2011 at California Pacific Medical Center in San Francisco because our company was based out there at that time. I want to reflect back on one of Marc’s comments because it was a really important one. In the early days, people said “Isn’t that going to be a necrotic device?” This is not a question anymore because we have been around long enough. Those gaps in the teeth allow for microperfusion of blood to the tissue. This was part of the evolution of Ovesco, as in the beginning, the approach to OTSC was pretty conservative. Hemostasis was not the driving factor. We were going after the most complex fistula cases because it was impossible to be there for bleeding cases. So, fistulae were the door opener for us to be in the GI suite because we could schedule these cases. Then the accidental perforations started kicking in, and now hemostasis is our fastest growing indication by far.

**MS:** The early days were like a classical startup. You have your product, your first prototype, and you run it through a series of animal labs. My background is in experimental surgery, so I had easy access to animal labs to study the device in terms of its efficacy in closing perforations, stopping bleeding, in Tübingen, Germany, where I am based. Then it was making the first real product. Initially, you do some outsourcing where you contract with vendors who make the product for you because you do not have those skills and resources. But I think one of the most important entrepreneurial decisions we made was to start our own in-house manufacturing. We did that very early on. In fact, we set up our manufacturing room in the basement of a neighboring residential apartment building near our original office. The floor was hand painted to make it washable and sealed by our first small group of people so that it could be used as a controlled manufacturing environment. We started making the complete device: the cap and the thread that is attached to it to release the clip. This evolved into our first clean room, which was built into a fire brigade house just across the corner. We now have a fabulous clean room here where we are making 30,000 of these OTSCs this year. Manufacturing brought us the skills, competence, and experience to build more variations because we became competent in this technology.

Our second most important step was to go direct in the United States. As a startup entrepreneur having a close friend, Geoffrey Yates, to partner with, we started presenting at conferences. People said it was cool and invited us to visit them. We were rather naïve because we had Geoffrey and we deployed one of our team to handle the East Coast. Basically, we visited whomever wanted to see us. It was a very basic way of getting started in the United States. It was not very strategic; it was really bottom up.

**GY:** I want to go back in time a bit more to share a bit more of the history. I met Marc in 1998 when I was a marketing guy at Boston Scientific and Marc was the consulting physician on the project I was assigned to, so our history goes back >24 years now. That is when he started his incubator back in the 1998 time frame, hiring a couple of engineers in Germany. Ten years later, I get the phone call, “Would you like to take the U.S. and run with it?” That was an easy decision to make. Our strategy was just me. So, our “strategy” was physicians would call, and I would grab the bag and go. I remember flying from San Francisco to Dayton, Ohio, and they said, “You came all the way over here for this?” That is what you do when you’re a startup. The next piece was using independent agents. We have moved away from that, and our U.S. team now is all direct. We also have 13 distributors in Latin America, for which I am responsible.

**LL: What is a clean room?**

**MS:** A clean room is a controlled environment with particle-free air where you do final assembly of medical products. You bring the parts into the clean room where people are dressed in special coats, and they assemble everything in there. The product is then packaged and sterilized.

Every OTSC and every derivative of OTSC that is used worldwide are made in Germany. We have some components shipped to us such as our caps, which are injection molded from plastic. We do not do injection molding. But every clip is made in house as we are a full-blown medical device operation. We are planning to start assembling OTSC systems in the United States this winter.

**LL: And will that be in North Carolina?**

**GY:** Yes, we bought a building in North Carolina 2 years ago and moved in last year. We have a clean room here with 2 clean room tents. We’ll be bringing the components from Germany and assembling them here in our clean room. We have a local provider to do packaging and sterilization. We will be rolling out our first limited batches in October.

**LL: What were the challenges you faced at Ovesco as you developed the OTSC?**

**MS:** Before we talk about the challenges, I wanted to share one of the more unique things about Ovesco. We have always been very clinical, and developing and maintaining relationships with clinicians has been a key priority. I am a medical doctor myself, so I understand how medicine works, clinical medicine is organized, what little time doctors have, and how many great ideas they have. I know how much they focus on clinical data, so clinical networking, being a good partner for our customers, and being really dedicated to clinical success is one of the key aspects. Doctors like you because you are an innovative player, and they want to see the professionalism in clinical education and clinical research. We have always given priority to running clinical trials, helping investigator-initiated trials by providing sufficient training. That has been a key priority for us. As a small company, this has not been easy for us. We have always been committed to supporting research in our products, to truthful scientific marketing, and to let the clinical results and benefits speak. Geoffrey, you always call that a clinical marketing process instead of a very commercial marketing process. That has been a key component in the journey of Ovesco as well as a key challenge because it requires a good amount of time.

**GY:** It is not easy building a medical device company from scratch. One company approached us many years ago and said, “Are you crazy, trying to do this on your own?” Companies are always talking to us, but we are not publicly traded. Therefore, we are not under the pressure of stockholders. We also are not venture backed because the venture guys want you to ramp it up as fast as you can, and then sell it to the Medtronics or Boston Scientifics of the world. That is not our strategy. We are privately held, privately owned. And here we are 13 years later in the United States and successful.

The regulatory pathways are challenging and even more so today, and it is not just regulatory pathways in the United States. It is the changes that are going on with the Medical Device Regulation (MDR) in Europe. We are getting ready to go live in Brazil in the next couple of months, and the regulatory challenges there have been daunting.

Navigating the hospitals with the value-added committees and the new product committees and vendor access—all those things make life more complicated. But companies that are successful find ways to navigate whatever terrain that they are encountering at the time. New product development is time-consuming and expensive. I like the way Marc phrased it: we are a very clinical sales- and marketing-driven company. I think that really resonates with physicians too. I have been involved in multiple product platforms throughout my career: OTSC has >500 publications on one product family, which is unheard of. This is not Ovesco: this is the physician community recognizing the importance of the technology and the positive outcomes they want to publish. We are really proud of that history.

**LL: I'm curious to understand your philosophy of why you are committed to being privately owned and independent, and what is driving that decision.**

**MS:** That is actually easy to explain because we have firm beliefs in what we do. We like what we do, and we believe we have a role in the medical device industry and in the clinical community that we would like to continue. Our organic growth has been 15% to 20% sales growth every year for >15 years. We are now about 150 people worldwide, with the majority in Germany but also growing in the United States, and now with subsidiary companies in India and France. It is easy to grow fast for 2, 3, 4 years but not for a decade. I believe we would not have been able to generate this consistent growth without a clear commitment to remain in our industry and to remain independent. For the employees, they may lose their jobs when you are acquired. The certainty that we are here to stay and saying this loud and clear has been a difference to what many other companies do. This has been possible because we are not venture backed. I provided funding, and we have a few private shareholders who came on board. We have now bought shares back from a few because they wanted to use their money for something else. We have been profitable for about a decade, and this organic growth is probably still the healthiest way to grow. I think our pretty steep growth of 20% a year depended a lot on our clear commitment to build ourselves as a responsible and innovative medical device company.

**GY:** For me, not being venture backed, we have been able to chart our own course in the way we feel is best for the company and for the physicians, our customers, and their patients. Again, I think that has been highly appreciated in the marketplace, the way we have approached it, and we are told that a lot. We could easily have sold out. That would have been easy to do years ago, and we probably could have gone faster if we had gotten a bunch of venture money, but then you are beholden to them to sell the company. We have done a building block approach, a brick at a time. I will add a new person as my bottom line allows me to do so.

In the United States, that is still our number one objective: to continue to build our commercial team in the field. When I say commercial, commercial to us is providing clinical support. The number one job of a representative in the United States is to support cases, train staff, and be there as a resource, not necessarily selling but being there as a knowledgeable resource. If they get a question they cannot answer, they get on the phone and find the answer immediately. I know that sounds really simplistic but that is what we do.

**LL: Would you discuss what your strategy has been in expanding globally?**

**MS:** Our formal strategy that we have had for about 10 years is encapsulated in 3 terms: intensify, enhance, and enlarge. By intensify, we want to intensify our reach to the customer. That means becoming more direct in sales representation because we believe we can represent our products the best ourselves. We have a dedicated proprietary sales force in Germany, Switzerland, France, Belgium, Luxembourg, and the United States. Of course, we cannot do that everywhere in all geographies. Therefore, we have trusted, longstanding distribution partners. Nevertheless, being close to our customers and intensifying customer contact is one of the elements in our strategy.

Enhancement means utilizing our existing technology platforms and improving them; using our platform to derive new applications. For example, converting OTSC into full-thickness resection device (FTRD) and stentfix OTSC. But the OTSC now is less than half the overall revenue pattern of the company. This comes from the enhancement aspect of combining things, innovating through combination, innovating through procedural innovation.

Enlargement means to build more technology platforms. We have an umbrella of digital products, telemetric devices such as our HemoPill product, which is a capsule for the diagnosis of upper GI bleeding. It is a hemorrhage-detecting sensor that can be swallowed, which is in the market here in Europe. We are still working on approval in the United States.

Therefore, our strategy is putting more emphasis on distribution, becoming more direct, rounding out our already established strength with the clipping technology, but also strategically adding new elements to it. It is actually not a very complex strategy and is relatively easily formulated. But how to implement it and how to convert this strategy into tangible results is the mission that is new every month and every year.

**GY:** On the enhancement side, I will also add that we are in a better position financially with resources and capacity, and that companies and physicians are approaching us now with ideas. We are going back and forth with a number of entities right now assessing new opportunities to add to our portfolio. Even though we might not have done the initial development, we want to have a deeper, broader, wider approach through the entire GI track. We are now in a much better position to do that, where we did not have the capacity to do that a few years ago.

**Figure 5:**
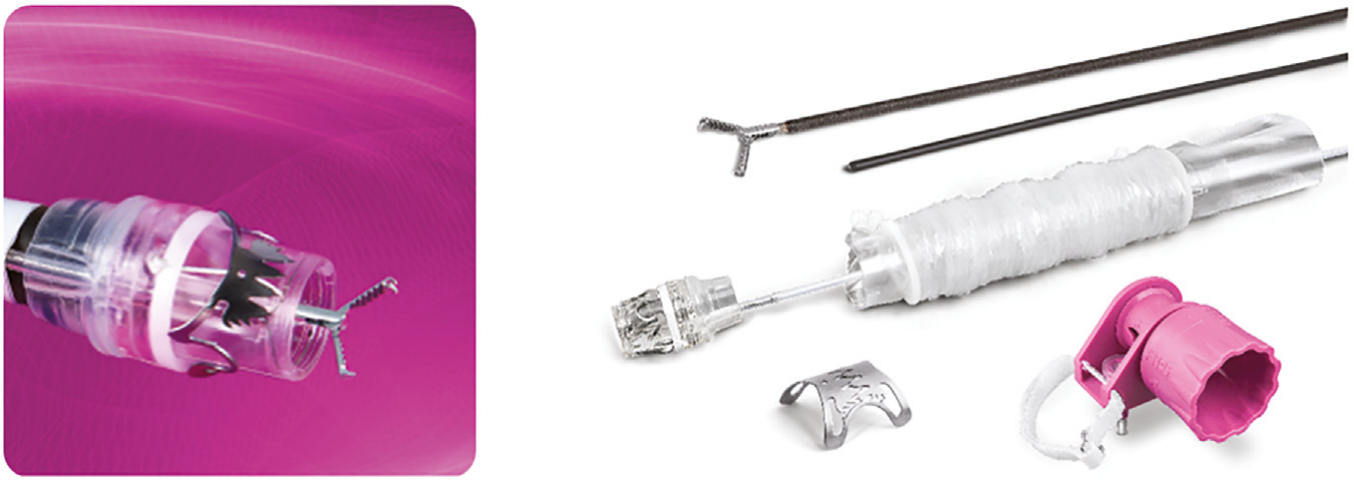
Full-thickness resection device.

**Figure 6:**
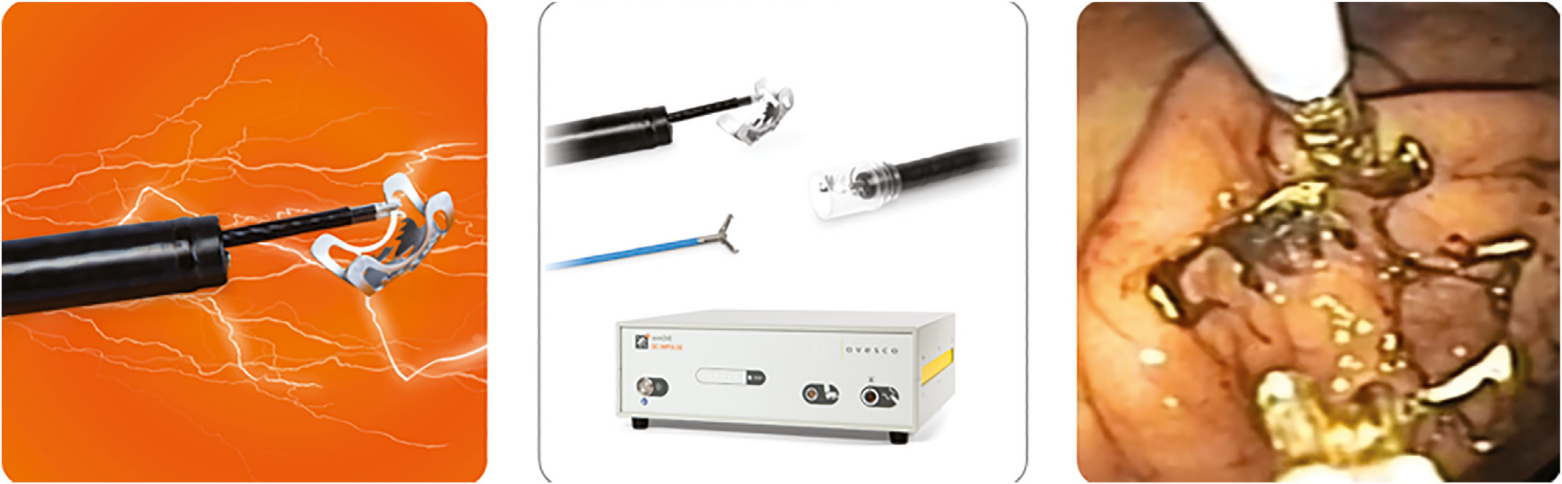
remOVE System (Ovesco Endoscopy AG, Tübingen, Germany) components and over-the-scope clip removal.

**LL: How do you decide what projects to work on next? (**Figs. 5 and 6**)**

**MS:** We like to take one step into the future and keep one leg on a secure position. This may involve progressing along the same indication where we are already present. For example, hemostasis is one of our most important indications, with numerous randomized controlled trials confirming the superior efficacy of OTSC in hemostasis. Therefore, being competent in hemostasis, why shouldn’t we become a hemorrhage detection company using novel technology?

Another approach would be to master a technology and bring that into new indications. An example of this is the whole clip derivative program by using the original clipping technology and opening up the market of bariatric endoscopy with our BARS device, which is a device for the reduction of enlarged gastrojejunal anastomosis in gastric bypass patients. Therefore, we either know the technology and feel secure and competent enough to explore a novel clinical field or we are competent in a clinical field and then add a new technology to it. That is a bit of the paradigm we use.

Otherwise, we do conventional assessment of business opportunities, market research, and networking as we have a very strong network in the academic scientific community. As Geoffrey said, they propose ideas, which we then jointly discuss. And not everything is strategic. Many things are just, we like it so we do it. We allow ourselves to be a little bit emotional.

**GY:** I think FTRD is a good example of how we move from one project to the next. The initial product was the colonic size, and the market needed something a little smaller for access. Then out of that, the device is being looked at now for full-thickness biopsy in the colon for Hirschsprung's disease. People were using our diagnostic device off-label in the upper GI tract, and it was not really suitable for that. So we designed, modified, and adapted that system for an FDA-approved gastroduodenal FTRD system, which is also being used at Brigham and Women’s Hospital in a clinical trial for gastroparesis. Therefore, you have that initial concept, and then we just keep taking it broader and wider.

**LL: We have discussed how there are many different types of OTSC and how some of these new ideas evolved organically. How long does it take from having the idea for modifying the clip to being able to use the new clip in humans?**

**MS:** If it is a derivative of our original technology, it is probably something that takes between 2 and 3 years. It could go faster, but the regulatory tightening that we are seeing is stopping us because it slows down innovation, especially in Europe with the MDR regulation and all the bureaucracy this has engendered. A really novel technology platform will take between 5 and 6 years, sometimes 7 years, because it goes all the way from discovery to implementation in prototypes and a lot of work in the laboratory with clinicians.

**LL: Would you explain the MDR in Europe and how it has impacted technology development? How is this process similar and different throughout the world?**

**MS:** The MDR is a new regulatory framework that the European Commission has set forth, and it took effect a few years ago. It is not necessarily a big change in the fundamental requirements of what you need to show to get your product CE marked or approved. But it has added a lot of bureaucracy to the essential steps. The documentation needs to be more formal, and the notified bodies also had to adapt to this new paradigm. Notified bodies are the entities that audit you and check if you are doing things properly. The new regulatory framework has kept them so busy that they simply do not have the time to completely recertify the whole medical device industry in Europe in a timely way. This is why everything takes so long. Therefore, getting a new product approved now takes 3 to 4 times as long and costs 3 to 4 times what it did 5 years ago.

Now has this process created value in terms of patient safety? Probably not. The increased costs have caused many companies to drop niche products. Doctors now complain that they are missing many niche products because companies have decided to drop the product because it is unbearable and costs too much. When a niche product is needed, there is often nothing else you can use. And that has created quite a bit of turmoil here in the clinical community. This does not apply to us as much but more to other companies that are smaller with special products that are not frequently used. The European Commission is critically reviewing this system because it is apparent that it produces more obstacles than it produces value.

One of the fundamental advantages of the FDA is that the FDA gives you a time window. When you submit, they are bound by law to evaluate your case within 90 days or whatever the framework is while notified bodies are not bound by any time window. This is why companies now tend to submit earlier in the United States because at least the FDA gives you a reliable time frame. That is a big asset, which in my opinion, attracts even more innovation toward the United States.

**GY:** Historically, CE marketing always happened about 2 years ahead of the U.S. approval. That window has certainly closed. I will say that the FDA is really good at giving you that 90-day window, but on day 90, they come back at you with a couple of questions and the clock starts ticking all over again. Every medical device company has to do this, and a lot of it depends on the type of product. If it is an accessory device or a line extension device, it is a lot easier as Marc said earlier. BARS and HemoPill have been challenging with a lot of questions and longer timelines. As a global company, we have to deal with these regulatory bodies all over the world, and they are all different. I referenced Brazil earlier. Their FDA is called ANVISA, and it has been brutal and expensive with question after question. But we have to do it as it is an important market. We are actually locked out of Canada right now because they have a requirement called MDSAP (Medical Device Single Audit Program). It is a classification like the International Organization for Standardization. They are the only country in the world that requires it, and we do not have it. We are going to get it, but the doctors are furious because our product registrations were pulled off the marketplace.

**MS:** The fundamental idea behind MDSAP is great where major markets in the United States, and I think Japan, Brazil, and Canada, have said they will accept a uniform audit that is done according to the standard. However, the United States, Brazil, and Japan still run their own national systems with Canada only accepting MDSAP as they shut down their local standard. This brings us back to the new European regulation system where to get an audit according to MDSAP for our company here in Germany will take years because the notified bodies do not have the capacity. I think Canada made a mistake here.

**LL: I know your company is very heavily invested in training and teaching; would you please talk about this work? What is the process you have been using for training clinicians?**

**GY:** I think it all starts with Marc. I have to give him the credit, and the best example is the FTRD. Every physician that utilizes FTRD has been through that training program and gets certified. When FTRD came along, the team in Germany formulated this all-day program because there is a lot going on with that device. There is a grasping device, a snare, scope manipulation, deployment, 6 hands involved. When you come to North Carolina, you have a didactic session and in the back of the building we have 4 endoscopic training towers. We have made that investment here in our facility. We use a porcine colon as the training model so you can feel the device and tissue. It is extremely detailed. Now that we have had experience over the last couple of years, we know everything that we need to know about FTRD. Our job during the training is to show you everything that can go wrong so we can prevent that from happening. We are not afraid to say that, and that is our job. A lot of the younger fellows who are coming out now, although they have had exposure to the system, they still come to get certified because they are going to another hospital somewhere else in the country. Then our field staff go in and train the nurses and the techs, support cases in person. Our representatives should be at every case they possibly can be at because that is what we do.

I also implore the physicians to have your A-team in the room, nurses and techs who like doing this kind of thing. That is the level of detail that we go into. Training is a cornerstone of our company. It is a major investment, and one of our largest expense lines. However, we have made that commitment and investment in training to attend every hands-on possible and bring physicians here. And it makes a difference. We know for a fact that our outcomes are better because of the training program.

**LL: What are the future plans for Ovesco? What things are you working on next?**

**MS:** We love what we are doing, and we enjoy the work. We have a strategy for Ovesco for the mid- and long-term future as being an independent player but highly networked, of course, and partnering with others. The independence allows us to continue innovating because we believe that is something that we do best. Much of Geoffrey’s and my role right now is to maintain the startup spirit in a company that is becoming bigger. We need to be more organized with more business units as we are international with subsidiaries, so everything needs to be more coordinated while we still strive at keeping our startup spirit.

We continue to grow into new areas of indication while keeping our stake in the existing ones to help improve interventional endoscopy with and for the clinical partners we are collaborating with and for the patients. This is a very nice mission to be on, making medicine even less invasive in the future by helping convert things that require surgery into endoscopic procedures. That is our mission. It is an intriguing mission, and we continue to grow. We have hired 20 people this year in Germany. Our mission attracts people from all professions, and they enjoy the purpose of the work at a medical device company.

**GY:** We are very passionate about what we do, and I think people see that when we are interviewing. All the people we are talking to comment how we are really passionate about what we do. I get a lot of gratification from our work helping physicians treat their patients, offering people employment opportunities and career growth. It is a wonderful combination. I am at the latter stage of my career where this is my last rodeo, and I would not continue to do it if I did not enjoy it.

Marc is absolutely right. When you hire someone new, they do not have an appreciation for what we did 10 years ago and how hard it was to get here. And it was really hard. We still struggle now, as the more people you add, the more difficult it gets. However, we have a unique chemistry. Our number one challenge always starts with the people, having the right people on our team that have that same passion and commitment to supporting you guys. This is what we are all about.

## Editor’s Closing Remarks

The partnership between physicians and industry has again led to innovations pushing the boundaries of what endoscopists have to offer patients. What began as an interesting research idea created the novel concept of OTSCs to close defects, treat bleeding, fix stents, and even perform full-thickness resections. Regulatory requirements around the world continue to pose challenges in promoting innovation and while they fulfill an absolutely essential role to ensure the safety of new devices, hopefully the processes will continue to be streamlined. Whether there will be a paradigm shift from conventional through-the-scope clips and thermal therapy to OTSCs in managing GI bleeding remains to be seen. There certainly remain challenges to using the larger device for both bleeding management as well as full-thickness resection, which requires careful training, preparation, and teamwork. Not only are the devices larger being over-the-scope but they also lead to tunnel vision, with difficulty negotiating tighter turns. However, if they can truly reduce recurrent bleeding in the real world outside of carefully managed trials, this is compelling in improving our care of patients with GI bleeding. Of course, it will be interesting to witness how the “bear claw” continues to reinvent itself to foray into other territories beyond closure and bleeding and helping propel the field of interventional endoscopy into new terrains.

## Disclosure

The following authors disclosed financial relationships: M. Schurr: Employee as Chief Executive Officer and shareholder for Ovesco Endoscopy. L. S. Lee: Consultant for Fractyl and Boston Scientific; research support and consultant for Fujifilm Medical. G. Yates: employee as President, shareholder, and board member for Ovesco Endoscopy USA.
